# Diabetes: Its Causes, Symptoms, and Treatment

**Published:** 1891-03

**Authors:** 


*Diabetes: its Causes, Symptoms, and Treatment.* By CHARLES W. PURDY, M.D. Philadelphia: F. A. DAVIS. 1890.

The book opens with an account of the distribution of diabetes in the United States: presenting as they do a large population with practically the same habits of life as regards dietary, &c., but living under great varieties of climate, observations in the States should give us valuable information as to the influence of climate on the occurrence of the disease. The author thinks that "cold and high altitude are the chief climatic features that determine a high mortality from diabetes": he acknowledges, however, that the statistics on which he bases his opinion are not so complete as could be wished, and further evidence seems to us to be necessary. Curiously, the Indians and Chinese resident in the United States do not suffer from the disease. The chapter on treatment is a useful one, and will be especially valuable to the American practitioner. The author has aimed at being concise; but although clearness and brevity are desirable in a manual of this kind, we think that a monograph on a particular disease should aim at giving complete information on the subject: if it contains no more and no less than the more advanced text-books and dictionaries on medicine, we fail to see its *raison d'être*. The author would do well in future editions to leave out some, if not all, of the clinical cases, which run to 46 out of a total of 173 pages, and are of no special value, and to make the general account of the pathology and symptomatology of the disease more full. There is a short chapter on diabetes insipidus, which adds nothing to our previous knowledge.

